# Mammarenaviral Infection Is Dependent on Directional Exposure to and Release from Polarized Intestinal Epithelia

**DOI:** 10.3390/v10020075

**Published:** 2018-02-10

**Authors:** Nikole L. Warner, Jenny D. Jokinen, Juliane I. Beier, Kevin J. Sokoloski, Igor S. Lukashevich

**Affiliations:** 1 Department of Microbiology and Immunology, University of Louisville Health Sciences Center, 505 South Hancock Street Rm 622, Louisville, KY 40202, USA; kevin.sokoloski@louisville.edu; 2 Center for Predictive Medicine for Biodefense and Emerging Infectious Diseases, Louisville, KY 40202, USA; jdjokinen@gmail.com (J.D.J.); j0beie01@exchange.louisville.edu (J.I.B.); 3 Department of Pharmacology and Toxicology, University of Louisville Health Sciences Center, Louisville, KY 40202, USA

**Keywords:** arenaviruses, mammarenaviruses, Lymphocytic Choriomeningitis Virus (LCMV), Mopeia Virus (MOPV), ML-29, intestinal epithelia, polarization

## Abstract

Mammarenavirusesare single-stranded RNA viruses with a bisegmented ambisense genome. Ingestion has been shown as a natural route of transmission for both Lassa virus (LASV) and Lymphocytic choriomeningitis virus (LCMV). Due to the mechanism of transmission, epithelial tissues are among the first host cells to come in contact with the viruses, and as such they potentially play a role in spread of virus to naïve hosts. The role of the intestinal epithelia during arenavirus infection remains to be uncharacterized. We have utilized a well-established cell culture model, Caco-2, to investigate the role of intestinal epithelia during intragastric infection. We found that LCMV-Armstrong, LCMV-WE, and Mopeia (MOPV) release infectious progeny via similar patterns. However, the reassortant virus, ML-29, containing the L segment of MOPV and S segment of LASV, exhibits a unique pattern of viral release relative to LCMV and MOPV. Furthermore, we have determined attachment efficacy to Caco-2 cells is potentially responsible for observed replication kinetics of these viruses in a polarized Caco-2 cell model. Collectively, our data shows that viral dissemination and interaction with intestinal epithelia may be host, tissue, and viral specific.

## 1. Introduction

Arenaviruses are enveloped viruses that have a single-stranded, bisegmented, ambisense RNA genome. The Large (L) segment, encodes the matrix protein (Z), and the RNA dependent RNA polymerase [1]. The Small (S) segment, encodes the nucleoproteins (NPs) and glycoproteins (GP) [1]. Initially, arenaviruses were thought to only infect mammals; however, recently arenaviruses have been identified as the causative agent of inclusion body disease in the boid family of snakes [2]. Hence, Arenaviruses have been separated into two genera on the basis of their natural reservoir hosts; mammarenavirus, which infect mammalian hosts, and reptarenavirus which infect reptilian host species [3]. Among the mammarenavirus genera, there is further subdivision into the Old World (OW) LCMV-Lassa virus complex and New World (NW), Tacaribe virus complex [3,4].

Lassa virus (LASV), the causative agent of Lassa fever (LF), is recognized as the most prevalent and most pathogenic of the OW arenaviruses. Annually in Western Africa, there are several hundred thousand clinical LASV infections, and thousands of deaths due to LF [5,6,7,8]. Although LASV causes a significant number of deaths, the majority of infections are apparently subclinical, or not severe enough to warrant emergency medical intervention, as over 45% of the population in endemic regions is seropositive for LASV; however, why some of the population develop disease and others do not it is not well understood [9]. Most recently, a study of almost 200 LASV sequences has shown that reservoir-to-human transmission is a primary driving force of LASV epidemics in Western Africa [10]. LASV is carried by its natural host *Mastomys natalensis*, and it is widely accepted that transmission of LASV to humans likely occurs via the ingestion of contaminated food-stuffs, or by the inhalation of infectious particles [5]. Indeed, an epidemiological study in the Republic of Guinea showed a link between the consumption of contaminated food as a risk for rodent-to-human transmission [11].

Due to its high lethality and transmissibility via aerosols, LASV is categorized by the Center for Disease Control and Prevention as a category A select agent. Currently, there are no clinically approved vaccines for LASV; and the antiviral drug ribavirin is the only treatment available for LASV infection. Nonetheless, caveats to this antiviral strategy/treatment regimen include severe side effects, and the requirement for early administration in order to have positive therapeutic effects [12]. Among limited vaccine candidates, only a LASV/Mopeia (MOPV) reassortant virus, ML-29, has been demonstrated to induce protective immunity against LASV strains from clade IV (Sierra-Leone, Liberia, Republic of Guinea) and clade II (Nigeria) [13,14,15,16,17,18,19]. Specifically, ML-29 is composed of the MOPV L segment, a non-pathogenic relative of LASV, and the S segment of LASV [20]. MOPV and ML-29 share the L RNA encoding L protein (RNA polymerase), and Z protein (matrix). Previous studies have determined that the L RNA segment of MOPV is the major factor of ML29 attenuation in vivo. Comparison of the ML-29 L segment with the parental MOPV L segment revealed the presence of numerous point mutations that may contribute to the attenuated phenotype associated with ML-29 [14]. While this reassortant has ML-29 specific mutations in the NP and GP2 proteins encoding by LASV S RNA, the attachment glycoprotein, GP1, is genetically identical to LASV.

Similar to LASV, the prototypic arenavirus Lymphocytic Choriomeningitis virus (LCMV), is genetically and biologically diverse. Transmission of this virus has been shown to share a similar mechanism to LASV, with transmission from rodents-to-non-human primates (NHP) and humans. Like the epidemiological study from the Republic of Guinea, a natural route of infection was observed within zoo kept tamarin populations, as animals that consumed LCMV-infected mice succumbed to LF-like illness and disease [21,22]. Importantly, these results have been recapitulated experimentally via the intragastric inoculation of NHPs with LCMV [23,24].

As mentioned earlier, LCMV strains are genetically and biologically diverse. LCMV-Armstrong (LCMV-ARM), a neurotropic strain, is highly adapted for infection in murine models. As such, exposure of NHPs to LCMV-ARM through either intravenously (i.v.) or intragastrically (i.g.) routes produced deeply attenuated sub-clinical infection [23,24]. In contrast, LCMV-WE has limited passage history in mice and tissue culture models of infection, and induced fatal LF-like disease in i.g. and i.v. infected NHPs, providing a surrogate model of LF at biosafety level (BSL)-3 containment [23,24]. Notably, infections via mucosal (i.g.) inoculation were attenuated during interaction with, and/or crossing the mucosal barrier of the gastrointestinal tract. Therefore, the intestinal epithelial are likely one of the natural gates of rodent-to-human transmission. Interestingly, when the LCMV-ARM or -WE i.g. infected NHPs were challenged with lethal doses of LCMV-WE intravenously, the animals did not succumb to LF-like disease as observed with PBS pretreated animals [24].

Due to the mechanism of transmission of arenaviruses from rodents to humans, epithelial tissue are among the first host cells to come in contact with the viruses, and as such they potentially play a decisive role in the spread of virus to naïve hosts [25]. The role of epithelial barriers on infection has been investigated extensively with a number of other viruses; however, the specific role of the intestinal epithelia on arenavirus infection remains to be exhaustively characterized [26,27,28,29]. Natural transmission via the intragastric route is generally considered to initiate with the interaction and infection of the epithelial cells from the apical side, whereas basolateral exposure of viruses requires damage, layer such as from a scratch or bite from an infected host, to the epithelial cell layer [30,31,32]. Here, we investigated the interaction of LCMV-Arm, LCMV-WE, Mopeia virus (MOPV), and the LASV/MOPV reassortant ML-29 with polarized Caco-2 intestinal epithelial cells, to investigate the role of barrier systems in viral dissemination, and to further elucidate the interactions of OW mammarenaviruses with the gastrointestinal epithelial. Collectively, our current studies support the model that viral dissemination and interaction with epithelia may be host, tissue, and viral specific.

## 2. Materials and Methods

### 2.1. Viruses and Titration Assay

VeroE6 (C1008) cells and Caco-2 (HTB-37) cells were purchased from American Type Culture Collection (ATCC) and grown in minimal essential media using Dulbecco’s modified eagle medium (Invitrogen, Carlsbad, CA, USA) containing 10% fetal bovine serum (Fisher Scientific, Hampton, NH, USA) and 1% antibiotic-antimycotic (Life Technologies, Carlsbad, CA, USA) in a humidified chamber at 37 °C under 5% CO_2_. Cells were infected with LCMV-Armstrong (strain 53b), LCMV-WE (strain 54), Mopeia virus (MOPV, strain AN20410), or the Mopeia/Lassa reassortant virus, clone ML-29 [33,34]. All viral stocks were generated using low multiplicity of infection (MOI) and stocks with titers ranging from 1 × 10^7^ PFU/mL to 1 × 10^8^ PFU/mL were stored at −80 °C until needed [17].

Viral titers were determined using a standard plaque assay with minor modifications [35]. Briefly, VeroE6 cells were seeded in the wells of a 12-well cell culture plate, and incubated until 80–90% confluent. Virus samples were serially diluted, and used to infect the Vero cells. Infection was carried out for 1 h in 37 °C. After this period, the cells were washed with DMEM without phenol red, and a semi-solid overlay containing 1X MEM, 5% FBS, and 0.5% Avicel (FMC BioPolymer, Philadelphia, PA, USA) (LCMV and ML-29) or 0.5% Agarose (MOPV). Cells were then incubated in a 37 °C humidified chamber with 5% CO_2_ for 5 days. The plaque assay cells infected with LCMV and ML-29 had the overlay media removed, were fixed with 4% paraformaldehyde solution for 15 min and cells were stained with 1% Crystal Violet solution to identify virus-infected cell foci. For titration of MOPV, virus-infected cells were covered with semisolid overlay of 0.5% agarose/5% FBS overlay. A 0.04% neutral red, 0.5% agarose, 5%FBS solution was added to wells after 4 days incubation. Both plaque assays have a limit of detection of approximately 80 PFU/mL.

### 2.2. Polarization of Caco-2 Cells and Infection of Polarized Cells

Caco-2 cells were seeded on 24-well plate Transwell inserts (Corning, New York, NY, USA) with a 0.45 micron filter as previously described [36,37]. Briefly, 160,000 cells in 0.5 mL were plated on “apical” side of insert, and 1 mL of nutrient media was added to each well. For basolateral seeding of the Caco-2 cells, the Transwell inserts were flipped upside down in a sterile container, and 0.5 × 10^6^ cells in a volume of 100 µL was placed on each Transwell surface. The cells were incubated for 1 h at 37 °C to allow for adherence to the membrane. Cells were then put back into the 24-well plate and 0.5 mL of nutrient media was added to each insert. Cells were maintained at 37 °C in 5% CO_2_ for 21 days until polarization was completed. Media was changed every 2–3 days with fresh media for the entirety of the polarization process. To determine if polarization of epithelial monolayers was complete, Transepithelial Electrical Resistance (TEER) was measured with an EVOM2 Epithelial Voltohmmeter (World Precision Instruments, Sarasota, FL, USA) as previously described [38]. In experiments with non-polarized Caco-2, cells confluent monolayers on day 3 after seeding were used. Tight junction proteins were analyzed with western blot (WB) and qRT-PCR. Antibodies for WB were obtained from the following: ZO-1 (61-7300), Claudin-1 (Thermo Scientific 51-9000, Waltham, MA, USA), Occludin (Thermo Scientific 71-1500). Pre-made primers/probe sets against human ZO-1 (Hs01551861) and Occludin (Hs00170162) were purchased from ThermoFisher.

Polarized Caco-2 cells were infected either apically, or basolaterally, with an MOI of 0.3 PFU/cell of the corresponding viruses: LCMV-Arm, LCMV-WE, MOPV, or ML-29. Wells and inserts were washed two times with DMEM without phenol red. To each insert, virus was added, and 1 mL of DMEM without phenol red was to each well. Infection was carried out for 1 h at 37 °C in 5% CO_2_. After this period, the inserts and wells were washed two times with 1x DPBS (Invitrogen), and 1 mL of nutrient media was added to each well and 0.5 mL was added to each insert chamber. Cells were maintained at 37 °C in 5% CO_2_. TEER was measured daily for 5 days, and the tissue culture supernatants from the Transwell inserts and wells were harvested daily and the media was replaced.

### 2.3. Confocal Microscopy

Caco-2 cells were grown apically on 12-well, 0.45 µM Transwell inserts (Corning) for 21 Days until a polarized monolayer was formed. Cells were then fixed with ice-cold methanol for 10 min at −20 °C, washed and stained on both the apical and basolateral sides of the inserts with antibodies against ZO-3 using monoclonal antibody against zonal occludin-3 (Cell Signaling, cat. # 3704, Danvers, MA, USA) at a 1:1600 dilution. Alpha-dystroglycan (α-DG) antibody clone 11H6C4, recognizing fully glycosylated α-DG, was used at a 1:100 dilution (Milipore, cat. # 05-593, Billerica, MA, USA). Hoescht 33342 (Thermo Fisher Scientific) was used for nucleus staining at 1:10,000 dilution was added for 10 min. Transwell filters were cut out and placed on microscope slides, followed by 10 uL of ProLong Gold (Life Technologies), and a cover slip placed on top. Slides were analyzed using an Olympus FV1000 laser scanning confocal microscope and analysis was done using IMARIS software (Bitplane, Version 7.7.1, Zurich, Switzerland).

### 2.4. Attachment Assay

Caco-2 cells were seeded and polarized for 21 days. Cells were infected with an MOI of 0.3 PFU/cell on either the apical, or basolateral, surface of polarized cells for all viruses. The cells were infected at 4 °C for 1 h to allow cells to attach to the cell surface, but not penetrate the cell. After the attachment period, the Input samples consisting of the cells and inoculum were directly harvested, and the experimental cells were washed several times with 1xPBS to remove unbound virus particles. Trizol-LS (Invitrogen) was used to harvest all of the aforementioned cells. RNA was isolated according to the manufacturer’s directions. Quantitative Real-Time Polymerase Chain Reaction (qRT-PCR) was used to quantitate attached viral particle. Primers and probe for ML-29/MOPV targeting the L segment: Forward (5′ TCCTCAATTAGGCGTGTGAA), Reverse (5′ TACACATCCTTGGGTCCTGA) and probe (5′ CCCTGTTCCCTCCAACTTGTTCTTTG). Primer and probe targeting LCMV-Armstrong L segment: Forward (5′ CCT TAA AGA GGT GAG AGC ATG A), reverse (5′ TTTCATTGATATTCTTGGTTAGGTG) and probe (5′ CAGCCACACCTGGATTCTGTAATTGG). Primer and Probe targeting LCMV-WE L segment: Forward (5′ CCT GGA CTC TGT AAT TGG CA), Reverse (5′ TTA CAT GCT CAG CAG CAC AG), and probe (5′ TCA CAG TGG ATT TCA CAC ACA ACC AGA).

The attachment of viral particles was assessed quantitatively via the ΔΔCt method [39]. Briefly, Ct values corresponding to the viral targets were normalized internally via the subtraction of the 18S rRNA levels detected within each sample. The resulting ΔCt values of the washed tissue culture cells were then compared relative to the bound unwashed Input controls. The resulting ΔΔCt values were used to determine the relative quantities of viral nucleic acids in the Bound (washed) and Input (unwashed) samples; these values were then plotted, and attachment was reported as further calculated via the ratio of Basolateral/Apical attachment.

### 2.5. Statistical Analyses

Statistical significance was analyzed using 3 biological replicates per experimental time point, using a Standard Student *t*-Test. All statistical values of *p* ≤ 0.05 were deemed as statistically significant.

## 3. Results

### 3.1. Infection of Polarized Caco-2 Cells with OW Arenaviruses Does Not Affect the Monolayer Integrity

**Figure 1:**
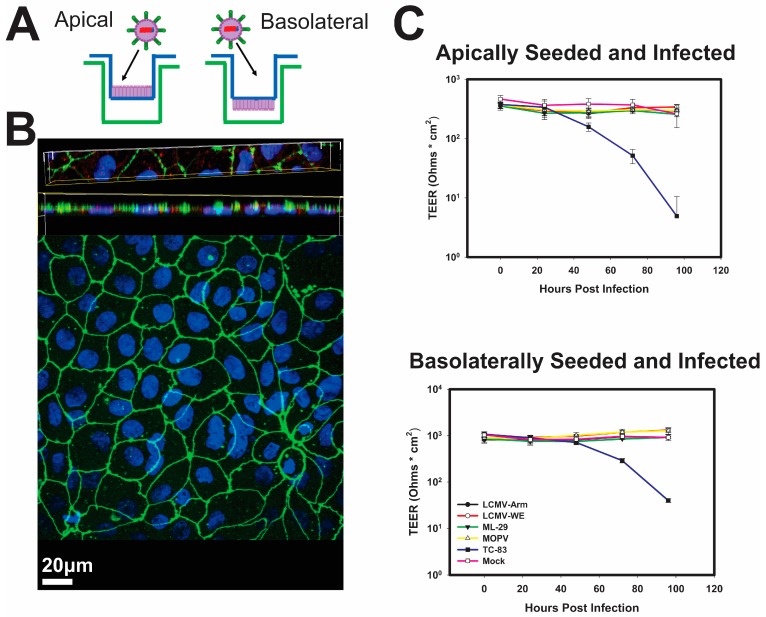
Old World (OW) mammarenaviruses do not alter integrity of model intestinal epithelia during infection. (**A**) Diagram of Caco-2 cell seeding for apical infection (left) or basolateral infection (right) during polarization; (**B**) After 21-day polarization period, Caco-2 cells form confluent monolayers with markers of polarization such as apical tight junction protein ZO-3 (green), and a-DG (red) on the basolateral side of cells; (**C**) Caco-2 cells were seeded for apical infection (top) or basolateral infection (below) and polarized for 21 days. After which the cells were either Mock infected with PBS, or lymphocytic choriomeningitis virus (LCMV)-Armstrong, LCMV-WE, ML-29, Mopeia (MOPV), or Venezuelan Equine Encephalitis (VEE) virus (vaccine strain TC-83) at an MOI of 0.3 PFU/cell. TEER measurements were taken daily for 5 days. Values shown are the means of 3 replicates, with the error bar representing the standard deviation of the means.

Since the gastrointestinal tract likely plays an essential role in the arenavirus rodent-to-human transmission, we used the human adenocarcinoma Caco-2 cell line [35] to establish an in vitro model of the intestinal epithelia lining of the gut (Figure 1A). The formation of a polarized monolayer was assessed by TEER and by detection of the tight junction protein Zonal-Occludin-3 (ZO-3), a partition marker of the apical and basolateral sides of the cells, was readily detected in between sister cells (Figure 1B). Furthermore, polarized Caco-2 were stained with antibody against α-DG, a principal cell receptor for OW arenaviruses (Figure 1B).

In general, the arenavirus species used in this study are not associated with cytopathic effects. Nonetheless, it was essential to the utility of our model to confirm that the apical and basolateral separations were intact during infection. In line with their non-cytopathic nature, the OW arenaviruses used in this study did not negatively affect electric resistance of epithelial monolayers during the 5-day observation period, suggesting that integrity of monolayers was preserved during the infection (Figure 1C). In contrast, the alphavirus Venezuelan equine encephalitis (VEEV) strain TC-83, which is known to be highly cytopathic, readily disrupted the integrity of polarized Caco-2 cells. Analysis of the mRNA and protein levels of tight junction proteins was tested and no significant change in quantity of tight junction proteins was observed in infected cells as compared to mock infected. 

**Figure 2:**
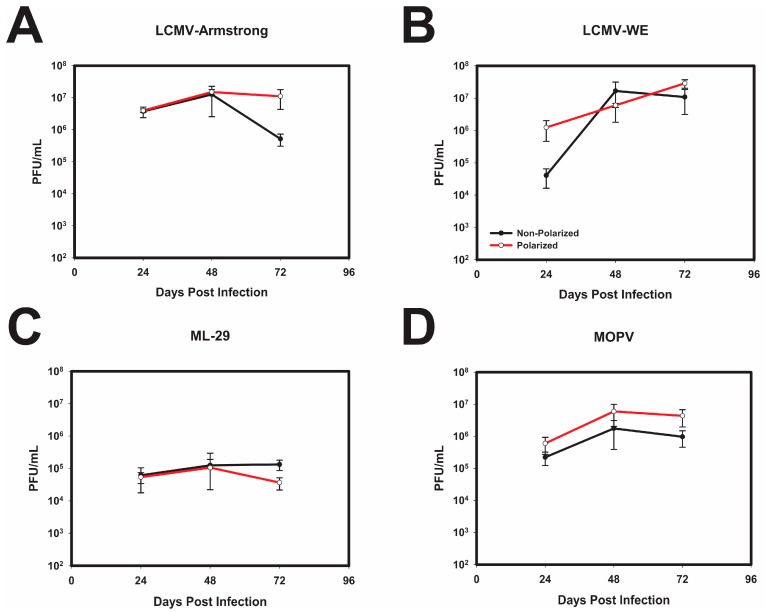
Polarization of Caco-2 cells does not significantly impact OW arenaviral replication. Caco-2 cells were seeded in 96-well plates and polarized for 2 weeks, or plated for 3 days (non-polarized) and infected with either LCMV-Arm (**A**); LCMV-WE (**B**); ML-29 (**C**); or MOPV (**D**) at a multiplicity of infection (MOI) of 0.3 PFU/cell. Supernatants were collected every 24 h for a 72 h period, and virus production was determined via standard plaque assay. Values shown are the means of 3 biological l replicates with the error bar representing the standard deviation of the mean.

To determine if cellular polarization unexpectedly perturbed the replication of OW arenaviruses, polarized and non-polarized Caco-2 cells were infected with both strains of LCMV, MOPV, as well as reassortant virus ML-29. The replication kinetics were monitored in both polarized and non-polarized cells by plaque assay. LCMV-Arm (Figure 2A) and LCMV-WE (Figure 2B) exhibited similar replication kinetics regardless of the polarization state of the Caco-2 cells. It should be noted that at 24 h post infection LCMV-WE did show a slight difference in titer between the polarized and non-polarized Caco-2 cells, but these differences were not observed in further time points. As such, neither LCMV-Arm, nor LCMV-WE, infections were significantly impacted by the polarization of Caco-2 cells during apical infection (Figure 2). In addition, similar to LCMV, ML-29 was not significantly impacted by polarization of these cells (Figure 2C); however, peak viral titers were approximately 2-log lower than those for LCMV or MOPV (Figure 2D). Taken together these results indicate that polarization of Caco-2 has minimal, if any, inadvertent effect on replication kinetics of the OW arenaviruses.

### 3.2. LASV/MOPV Reassortant ML-29 Exhibits Different Viral Entry and Exit Patterns Compared to Either LCMV or MOPV

As described earlier, the gastrointestinal tract is one of the major gates of arenavirus entry during rodent-to-human transmission [5,10,11]. During transmission, epithelial barriers may affect pathogenicity of the OW arenaviruses [23,40]. To assess the role of the intestinal epithelial barrier during infections of LCMV strains with different pathogenic potential for NHPs, the polarized Caco-2 cells were exposed either apically or basolaterally to the aforementioned OW arenaviruses. To verify the integrity of the polarized monolayer during the experiment, TEER was measured regularly, and the apical and basolateral supernatants were collected every 24 h for a period of 5 days.

**Figure 3:**
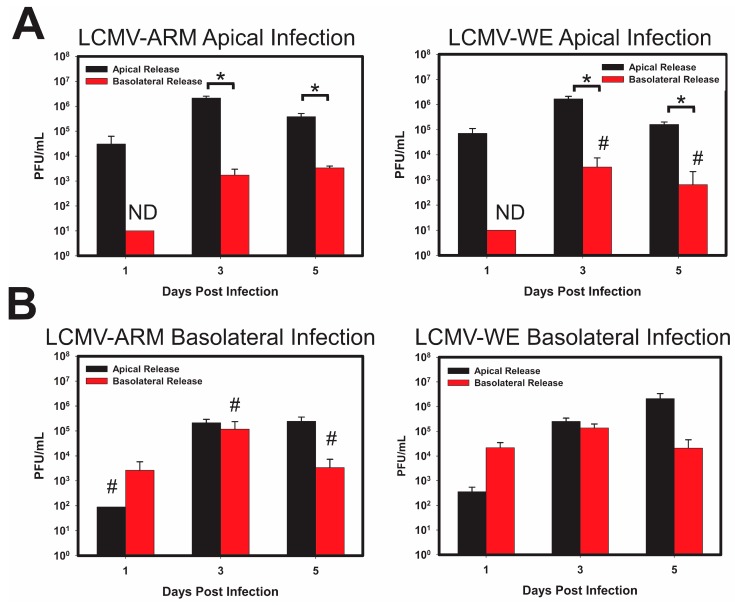
LCMV-Armstrong and LCMV-WE show similar patterns of entry and release in polarized Caco-2 cells regardless of pathogenic differences. Caco-2 cells were polarized on 0.4 µm Transwell inserts in apical or basolateral orientation, for 21 days. After integrity of the monolayer was verified using TEER, the cells were infected with either LCMV-Arm, LCMV-WE, at an MOI of 0.3 PFU/cell on either the apical (**A**); or basolateral (**B**) side of polarized Caco-2 cells. Supernatants were collected from both the insert, and well of the Transwells, to determine viral release from the apical or basolateral surfaces independent from one another. Viral titer was measured using standard plaque assay. Release from the Apical surface (black) and the Basolateral surface (red) is plotted with respect to time, with initial viral load subtracted. Values shown are the means of 3 biological replicates with the error bar representing the standard deviation of the mean. If viral plaque forming units (PFUs) were not observed, data received a place-holder value to signify samples were tested, but no data (ND) was collected. # indicates that one or more biological replicates was below limit of detection. * *p*-value ≤ 0.05.

Apical exposure of polarized Caco-2 cells to both strains of LCMV resulted in robust infection and virus release from primarily the apical cell surface. Nonetheless, basolateral release of infectious virus particles was observed at later times post infection. Therefore, while infectious particles were released from both surfaces, the release was more efficient from the apical surface, with a ~2-log difference between the two supernatants (Figure 3A). In contrast, when polarized Caco-2 cells were exposed to LCMV-Arm and LCMV-WE via the basolateral side, infection resulted in roughly the equivalent release of infectious particles from both cell surfaces (Figure 3B).

**Figure 4:**
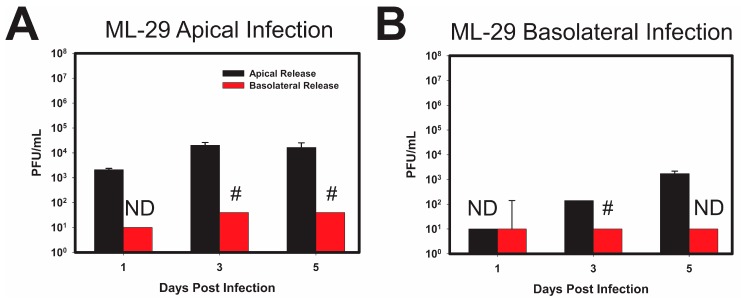
ML-29 replication in polarized Caco-2 cells differ from the replication patterns of LCMV. Caco-2 cells were polarized on 0.4 µm Transwell inserts as stated for Figure 3. Cells were then infected on the apical (**A**) or the basolateral (**B**) surface of the cells with an MOI of 0.3 PFU/cell of reassortant vaccine candidate ML-29. Supernatants were collected from transwells and inserts to determine viral release from the apical or basolateral surfaces independent of one another, on every day for 5 days. Release from the Apical surface (black) and the Basolateral surface (red) is plotted with respect to time, with initial viral load subtracted. Values shown are the means of 3 biological replicates with the error bar representing the standard deviation of the mean. If viral PFUs were not observed, data received a place-holder value to signify samples were tested, but no data (ND) was collected. # indicates that one or more biological replicates was below limit of detection. * *p*-value ≤ 0.05.

In addition to the two strains of LCMV described above, the patterns of viral entry and release of ML-29 was assessed in the polarized Caco-2 model. As shown in (Figure 4A) Caco-2 cells apically infected with ML-29 failed to produce detectable virus particles from the basolateral surface, despite the apparent release of infectious viral particles from the apical side. Comparative analysis indicates a 2-3-fold difference between viral apical and basolateral release during apical ML-29 infections of polarized Caco-2 cells. Curiously, infection of the polarized Caco-2 cells via the basolateral side resulted in only apical release (Figure 4B). Notably, the release of infectious ML-29 progeny were temporally delayed during basolateral infections, and resulted in the formation of low viral titers.

**Figure 5:**
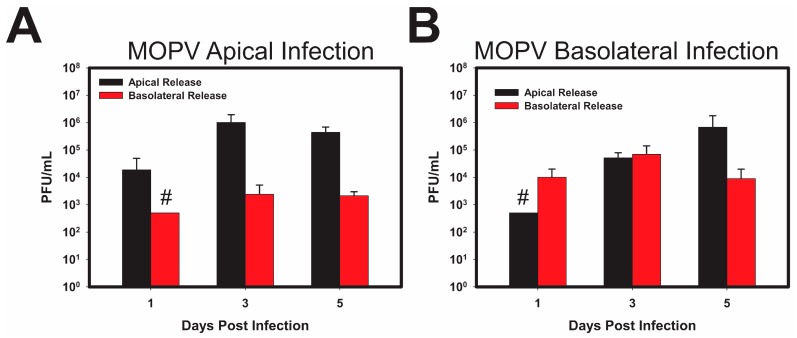
Mopeia Virus replication in polarized Caco-2 cells follows a similar pattern as LCMV replication. Caco-2 cells were polarized on 0.4 µm Transwell inserts in apical or basolateral orientation, for 21 days. After integrity of the monolayer was verified using TEER, the cells were infected with MOPV, at an MOI of 0.3 PFU/cell on either the apical (**A**) or basolateral (**B**) side of polarized Caco-2 cells. Supernatants were collected from both the insert, and well of the Transwells, to determine viral release from the apical or basolateral surfaces independent from one another. Viral titer was measured using standard plaque assay. Release from the Apical surface (black) and the Basolateral surface (red) is plotted with respect to time, with initial viral load subtracted. Values shown are the means of 3 biological replicates with the error bar representing the standard deviation of the mean. If viral PFUs were not observed, data received a place-holder value to signify samples were tested, but no data (ND) was collected. # indicates that one or more biological replicates was below limit of detection

Parallel analysis of MOPV infection reveals a pattern of viral release similar to that observed for LCMV. As shown in (Figure 5A), the apical infection of polarized Caco-2 cells primarily resulted in the release of infectious viral particles from the apical surface; however, basolateral release was observed. Similar to LCMV, and different from ML-29, basolateral infection of polarized Caco-2 cells resulted in the more-or-less equivalent release of viral progeny apically and basolaterally.

### 3.3. Attachment and Binding of OW Arenaviruses to Polarized Caco-2 Cells

To assess if the aforementioned OW arenaviruses differed in their capacity to attach/bind to intestinal epithelia, polarized Caco-2 cells were infected at an equal MOI (0.3 PFU/Cell), via the apical or basolateral surfaces. Cells were incubated at 4 °C for 1 h to allow virus to attach, but not penetrate the host cell. To determine the relative attachment rates of the viral particles, the total RNA was extracted from unwashed and washed tissue culture cells of at least three biological replicates derived from at least two independently generated viral stocks. The relative abundance of viral RNA was detected by qRT-PCR using virus-specific primers to determine the percent of input of virus particles that bound to the polarized Caco-2 cells.

**Figure 6:**
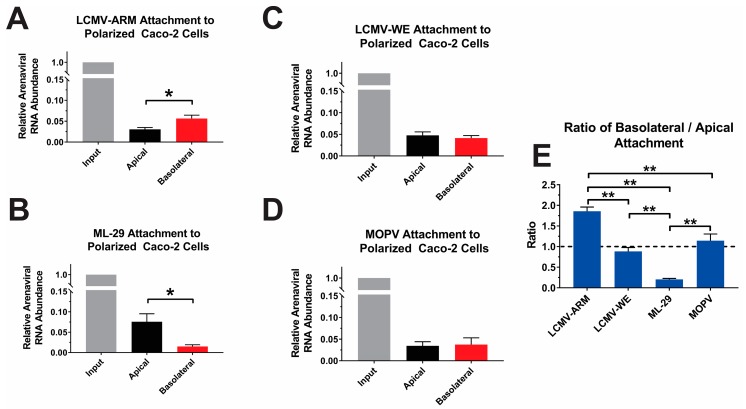
Attachment of ML-29 on the basolateral surface of polarized Caco-2 cells is significantly lower than apical attachment. Caco-2 cells were polarized on 0.4 µm Transwell inserts for 21 days in either apical or basolateral orientation. Cells were then infected with an MOI of 0.3 PFU/cell of LCMV-Arm (**A**); LCMV-WE (**B**); ML-29 (**C**); or MOPV (**D**) at 4 °C for 1 h to allow virus to attach to the epithelial cell surfaces, but not enter the cells. After 1 h, cells were either unwashed (input) or washed and cellular RNA and supernatants were collected for quantitative real-time polymerase chain reaction analysis. Ratio of basolateral to apical attachment was taken by dividing basolateral ΔΔCT values of washed cells by the apical ΔΔCT values of washed cells (**E**). * *p*-value ≤ 0.05, ** *p*-value ≤ 0.01.

Analysis of LCMV attachment indicated that for both LCMV-ARM and LCMV-WE ~5% of the input virus adsorbed to the polarized Caco-2 cells (Figure 6A,B). Comparisons of apical- and basolateral-bound viral levels indicated that LCMV-ARM exhibited preferential binding to the basolateral surface of the polarized Caco-2 cells (Figure 6A). LCMV-WE, in contrast, did not exhibit preferential binding to either surface (Figure 6B). Collectively, these data indicate a potential difference between the two LCMV strains in regards to cell attachment. Assessment of ML-29 binding indicated a strong attachment preference to the apical surface of the cells. As shown in (Figure 6C), approximately 4-fold more virus attached to the apical surface relative to the basolateral surface of polarized Caco-2 cells. These data are in apparent congruence with the observations reported in Figure 4, where basolateral infection were less efficient as compared to parallel apical infections. Quantitative analysis of MOPV attachment reveals similar observations to LCMV-WE. Specifically, as shown in (Figure 6D) the attachment of MOPV virus was equivalent amongst the apical and basolateral surfaces.

Since we cannot directly compare the absolute levels of attachment for each surface between the individual virus species, we must compare the ratios of apically and basolaterally bound viruses to identify statistical differences between the four OW arenaviruses used in this study. Overall, these comparisons indicate that LCMV-ARM and ML-29 differ from the other viruses, and each other, in regards to their binding proclivities. LCMV-Arm has a ratio of apical and basolateral attachment of approximately 1.5, indicating that attachment has a slight preference to the basolateral surface of the polarized Caco-2 cells (Figure 6E). The ratio of basolateral and apical attachment of LCMV-WE and MOPV of approximately 1, indicates that LCMV-WE and MOPV attachment is more or less equivalent in its binding to the apical and the basolateral surfaces of Caco-2 cells (Figure 6E). While LCMV-ARM is unique in its binding preference as compared to the other viruses, this indicates that LCMV-ARM, LCMV-WE, and MOPV all attach to some degree on the apical and basolateral surface of the cells. However, the ratio of basolateral and apical attachment of ML-29 indicates that there is a large difference between viral attachment between the two surfaces, with a significant preference for the apical side of the polarized Caco-2 cells (Figure 6E). ML-29’s preference for the apical surface with comparatively little to no binding on the basolateral surface, is significantly different than that of LCMV and MOPV. Collectively, these data indicate that LASV-GP has a significantly different binding efficacy to the basolateral side of Caco-2 monolayers as compared to LCMV and MOPV.

## 4. Discussion

Epidemiological observations in West Africa indicate that the ingestion of food contaminated with excreta of infected *M. natalensis* is one of the natural mechanisms of LASV transmission to humans [10]. Up to 45% of individuals living in some LASV endemic regions in Western Africa can be seropositive to the virus; and if re-infection occurs, seropositive individuals can protect themselves from disease onset. However, seronegative individuals may also be protected from disease, due to protection associated with cell-mediated immunity [41]. This implies high prevalence into endemic populations; however, the precise mechanisms behind this phenomenon are unknown. In NHP studies of arenavirus disease, LCMV-WE causes LF-like disease via intravenous (i.v.)-infection, whereas LCMV-ARM shows chronic infection, with no disease onset [23]. Furthermore, high titer i.v. infections of NHPs with LCMV-WE resulted in uniform mortality [23]. Nevertheless, i.g. infection with identical titers showed a variable outcome, ranging from no signs and symptoms to fatal, LF-like disease with elevated aspartate and alanine aminotransferases (ALT/AST) levels [23,24]. Elevated ALT and AST levels are symptomatic of hepatic tissue damage, and highly elevated levels are associated with lethal LF disease of humans in endemic regions [42]. In addition, some of the surviving animals survived i.v. challenge, indicating that LCMV infection did occur as a competent humoral response was induced [24]. Due to probable infection via the ingestion of contaminated food stuffs, and the variable outcomes of intragastric infection in vivo, we sought to investigate whether or not the patterns of entry and exit when polarized Caco-2 cells were exposed to OW mammarenaviruses apically (intragastric route) or basolaterally (intravenous route).

As previously described, LASV and LCMV entry into MDCK cells and HBE cells occurs primarily via the basolateral side, and release of viral particles was predominantly from the basolateral surface. These in vivo and in vitro studies led us to investigate the role of the intestinal epithelial barrier during OW arenavirus infection. In this study, we characterized an in vitro model of intragastric infection to assess the interaction of OW mammarenaviruses with the intestinal epithelia in an amenable tissue culture system. This system utilized polarized Caco-2 cells grown on transwells, which is a well-established cell type and cell culture system used for in vitro studies of the intestinal barrier [36,38]. This system enables the independent examination of the role of the apical and basolateral epithelia surfaces during viral infection. Therefore, this model recapitulates the infection of intestinal epithelial cells from the luminal and laminal sides of the epithelial monolayer via the apical and basolateral surfaces, respectively.

Using this model system, we evaluated infections of LCMV strains of different pathogenic potentials. We also used a MOPV/LASV reassortant, ML-29, a validated BSL2 surrogate model that is capable of mimicking the interaction of LASV with susceptible cells. ML-29 expresses GP1 attachment glycoprotein identical to LASV GP1, and MOPV, attenuated genetic relative of LASV. Since ML-29 is a reassortant virus of LASV/MOPV, it represents a better system in which arenaviral biology can be determined compared to VSV or retrovirus-based pseudotypes expressing LASV GPC as the viruses contain genuine arenaviral replication machinery.

From the in vivo studies in NHPs, it was expected for virus to release infectious particles from primarily the apical side, since viral particles were not detected in any tissues after intragastric infection with LCMV [23]. However, when exposed on the apical side of epithelial cells, LCMV and MOPV primarily released on the apical side of the cells, but release basolaterally was observed. This observation indicates that the epithelial barrier is not the sole determinant of viral dissemination. Interesting to note as well, was that patterns of replication were similar regardless of the in vivo pathogenicity of the LCMV strains. Therefore, the infectious capacity of these viruses in vitro, in regards to Caco-2 cells, does not correlate with pathogenic properties for LCMV. Therefore, further investigation as to the driving forces of pathogenic differences needs to be investigated. However, in contrast to LCMV, the ML-29 expressing LASV GP1, predominantly released viral particles apically regardless of the route of entry. Furthermore, these results demonstrate that MOPV entry-release pattern in polarized Caco-2 cells resembled those in cells infected with LCMV, and was clearly different from ML29-driving entry-release. Due to ML-29 replication patterns being different than that of MOPV replication, these patterns of entry and release are not due to MOPV replication machinery, and may be attributed to the gene products of the S segment of LASV, namely the glycoproteins and nucleoprotein. This poor replication and egress to the basolateral sides of the cells is an interesting observation. While ML-29 is not WT-LASV, using WT-LASV in similar studies could lead to an explanation as to why almost half of the population of endemic regions are seropositive for LASV, but never demonstrated clinical signs of disseminated illness. In addition, the data presented here indicates that the capacity to infect via the apical surface of intestinal epithelial cells is not a primary determinant of arenavirus pathogenesis.

ML-29 was developed as a potential vaccine for the prevention of LASV infection. As seen here, ML-29 had a much greater binding rate leading to successful infection when exposed to the apical side of polarized intestinal epithelia. If after successful attachment following the ingestion of viral particles fails to release viral particles basolaterally, this may be an exceptionally potent tool for the development of successful immunity against LASV in at-risk populations. These results provide additional evidence for attenuation of ML-29 as a vaccine strain for LASV. From these in vitro studies, and the dire need for a LASV vaccine, studies examining the importance of the route of exposure to ML-29 in an in vivo model should be investigated as a potential therapy to LASV infection and prevention.

A recent publication from Oppliger et al. identified entry of a recombinant LCMV expressing LASV-GP (rLCMV-LASVGP) in polarized Caco-2 cells [43]. Here, rLCMV-LASGP showed preferential entry into polarized Caco-2 cells on the basolateral surface of these cells. Opposingly, we identified via qRT-PCR that viral attachment of ML-29, a reassortant containing the GP1 of LASV, preferred the apical surface of polarized Caco-2 cells. Interestingly, we did see LCMV preferentially attaching to the basolateral surface of polarized cells, as seen with rLCMV-LASVGP. However, the rLCMV-LASGP studies did not elucidate the viral release patterns of rLCMV-LASVGP in polarized Caco-2 cells, nor the initial attachment of viruses to these cell surfaces. Furthermore, due to ML-29’s genetic differences to the LCMV-backbone of rLCMV-LASVGP, our results and those of Oppliger et al. cannot be directly compared. Taken together, our observations, are a useful addition to the field to further investigate precise differences between rLCMV-LASVGP and ML-29 in order to evaluate genetic variations of these viruses to more accurately identify potential targets for LASV therapeutics and further understand the replication cycle of LASV.

Some questions still lie as to the reason that ML-29 bound so inefficiently on the basolateral side of Caco-2 cells. Although primary receptor (α-DG) is located on the basolateral surface of the polarized Caco-2 cells, this data, along with the data of others, provides further support that LASV has complex receptor usage. Additional receptors for LASV should be investigated in the Caco-2 system, including Axl, DC-SIGN and Tyro3. Along with additional receptors and their role in our system, the interaction with α-DG should also be investigated. An excellent review by Torriani et al. describes a number of studies that explain the complex viral-receptor interaction of LASV [44]. Although fully functional α-DG was detected basolaterally in polarized Caco-2 cells, ML-29’s binding efficiency was low. This may be due to a multitude of reasons including the use of other cellular factors and receptors used in addition to α-DG to attach to these polarized cells, or differences and mutations that ML-29 may contain as compared to WT LASV, especially those present in the GP2 protein. Previous research has identified that LASV infection was dependent upon sodium hydrogen exchangers (NHEs), as well as actin cytoskeleton to have successful viral entry into host cells [45]. Investigations into these factors during infection of polarized Caco-2 cells should be analyzed to determine a reason for inefficient and poor binding of ML-29 on the basolateral side of these cells. Although ML-29 contains the S segment of LASV, the L segment of MOPV may interfere with complete and successful viral replication that WT LASV may have, comparatively. However, we believe the latter to be a minimal or insignificant inhibition of viral attachment and replication due to MOPV replicating similarly to LCMV in polarized Caco-2 cells when exposed to the basolateral side of the cells, as compared to little or no viral replication by ML-29 after basolateral exposure. Thus, investigation into precisely how WT LASV attaches, enters, and releases from polarized Caco-2 cells would be a valuable addition to the field.

## 5. Conclusions

To conclude, our data above demonstrates that the polarized Caco-2 system is a viable model to investigate the interaction of intestinal epithelial cells during viral infection with OW mammarenaviruses. These polarized epithelia closely mimic intestinal epithelial of human hosts and allow further investigation of mammarenaviral infection at the epithelial barrier. These data with LCMV show that intestinal epithelial cells may not be the sole determinant of viral pathogenesis and dissemination. Furthermore, differences between prototypic arenavirus LCMV and the surrogate model of LASV interaction, ML-29, were observed in both attachment efficiency and viral entry and egress from polarized intestinal epithelia. These results may potentially explain the high penetrance without disease observed for LASV. In addition, ML-29’s diminished binding efficiency to the basolateral side of polarized Caco-2 cells supports the expanding complexity of arenavirus receptor interactions. Collectively, these studies show that arenaviral infection of polarized cells is not only viral specific, but ultimately may be tissue and host-specific as well; and that arenavirus infection and pathogenesis may be dependent on asymmetric distribution of viral and cellular factors required for virus entry and budding.
